# Orchestrating serine/threonine phosphorylation and elucidating downstream effects by short linear motifs

**DOI:** 10.1042/BCJ20200714

**Published:** 2022-01-06

**Authors:** Johanna Kliche, Ylva Ivarsson

**Affiliations:** Department of Chemistry – BMC, Uppsala University, Husargatan 3, Box 576 751 23 Uppsala, Sweden

**Keywords:** kinase, modular domain, phosphatase, protein–protein interactions, SLiM

## Abstract

Cellular function is based on protein–protein interactions. A large proportion of these interactions involves the binding of short linear motifs (SLiMs) by folded globular domains. These interactions are regulated by post-translational modifications, such as phosphorylation, that create and break motif binding sites or tune the affinity of the interactions. In addition, motif-based interactions are involved in targeting serine/threonine kinases and phosphatases to their substrate and contribute to the specificity of the enzymatic actions regulating which sites are phosphorylated. Here, we review how SLiM-based interactions assist in determining the specificity of serine/threonine kinases and phosphatases, and how phosphorylation, in turn, affects motif-based interactions. We provide examples of SLiM-based interactions that are turned on/off, or are tuned by serine/threonine phosphorylation and exemplify how this affects SLiM-based protein complex formation.

## Introduction

Cellular function relies on interactions between globular domains and short linear motifs (SLiMs) that are commonly found in the intrinsically disordered regions of the proteome [1]. SLiMs bury 3–4 residues in the binding pocket of their folded binding partners, and the affinities are typically in the low-to-mid micromolar range. The affinity and specificity of these interactions may be increased by avidity effects from arrays of motifs binding to multidomain proteins and by cellular localisation and temporal regulation of expression [2]. Moreover, the interactions are frequently regulated by post-translational modifications (PTMs) that may create or break motif binding sites or tune affinities [3]. Among the PTMs, protein phosphorylation is the most frequent modification, and is the focus of this review.

Phosphorylation occurring on serine, threonine and tyrosine residues (the O-phospho-proteome) has been most extensively studied. In addition, N-linked (histidine, lysine, arginine), A-linked (aspartic and glutamic acid) and S-linked (cysteine) phosphorylation have also been reported and added to the versatility of this PTM [4]. Mass spectrometry-based approaches have identified hundreds of thousands of phosphosites [5,6]. Reports on phosphosites have been collected in resources such as PhosphoSitePlus [7], PhosphoNET [8] and phospho.ELM [9]. Efforts have also been directed towards deciphering the substrate specificity of kinases which catalyse phosphorylation, and several kinase-phosphosite predictors have been developed [10–12]. Similarly, systematic attempts have been made towards elucidating the substrates of human phosphatases, that catalyse the reverse reaction [13], and available information has been gathered in the DEPOD database [14]. However, most phosphosites remain orphan in terms of which kinase acts on them, and which phosphatase dephosphorylates them. Another open question is which of the reported phosphosites are of functional relevance for the regulation of cellular functions. Resources, such as phospho-ELM [9] and switches.ELM [3], give an overview of the reported modulation of SLiM-based interactions by phosphorylation. Nevertheless, our understanding of how phosphorylation affects those interactions remains fragmented, especially given that most of them are likely yet unknown.

**Figure 1.**
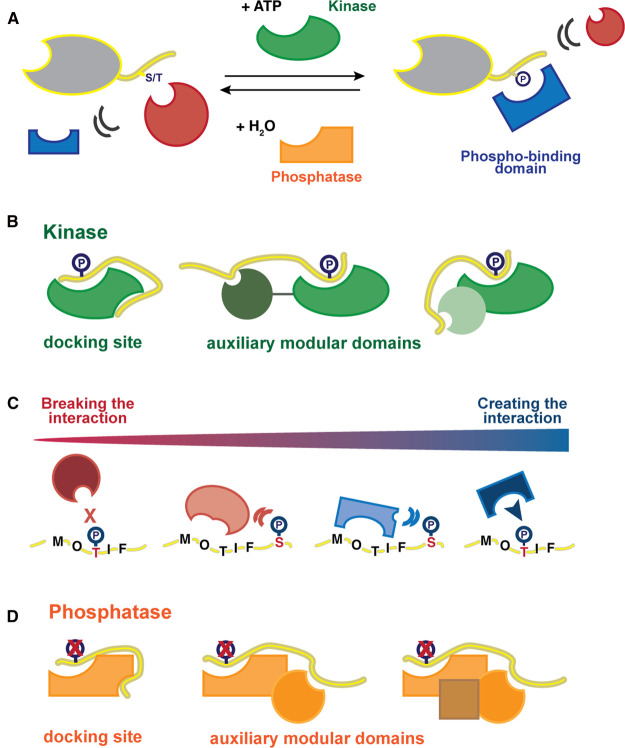
Overview of the system of readers, writers, and erasers of protein phosphorylation. (**A**) Schematic of the phosphorylation event impacting PPIs, either creating (blue phospho-binding domain) or breaking (red non-phospho-binding domain) a given interaction. The writer of the phosphorylation code is the kinase (green) and the eraser is the phosphatase (orange). (**B**) Kinase docking motifs can bind to binding sites on the kinase outside of the catalytic site, or to auxiliary domains that are either linked to the kinase domain, or are independent regulatory protein moieties associated with the catalytic domain (for example cyclins). (**C**) Phosphorylation on core motif residues can create (blue) or break (red) interactions, whereas phosphorylation of flanking or interspersed residues of the motif may result in modulation of affinities. (**D**) Serine/threonine phosphatase complexes are often composed of multiple subunits that can include a catalytic domain, a regulatory domain, and a scaffolding domain. Docking motifs can bind directly to the catalytic domain, or to docking sites on other domains of the complex.

In this review, we highlight the roles of SLiMs in orchestrating serine/threonine phosphorylation and dephosphorylation, and how motif phosphorylation alters interactions between binding partners (Figure 1A,C). We focus on the docking motifs that contribute to the targeting of serine/threonine kinases and phosphatases (Figure 1B,D) to their substrates, and the effects of phosphorylation on SLiM-based interactions (Figure 1C). We use the term docking interactions in a broader sense for SLiM-based interactions that contribute to target the enzymes (kinases or phosphatases) to their substrates.

We do not attempt to cover the literature comprehensively but use representative examples and recent studies as illustrations, as well as highlighting the functional consequences on different cellular processes.

## The writers — SLiM-based interactions that target kinases to their substrates

**Figure 2.**
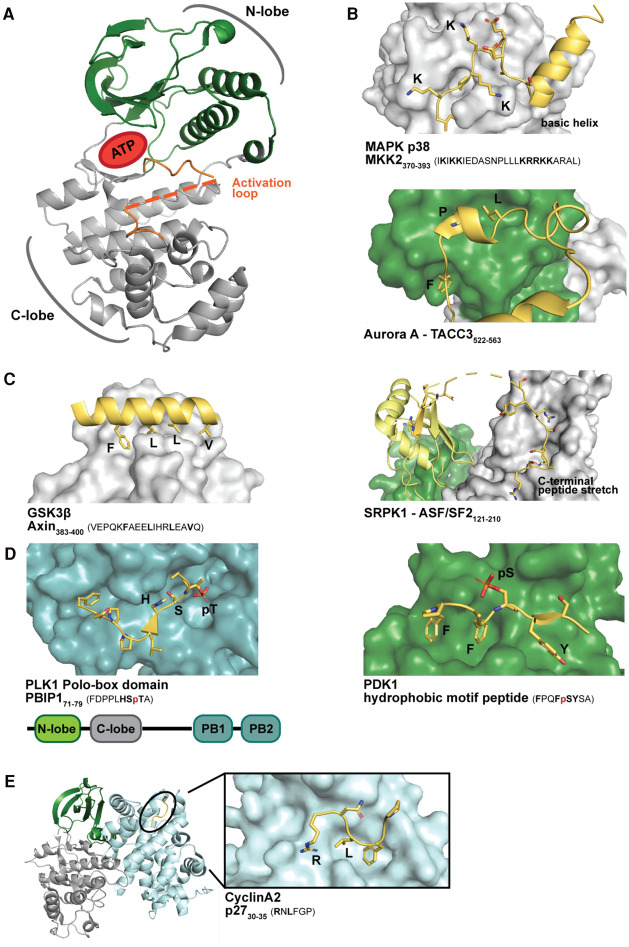
Strategies of kinases for substrate targeting by making contacts outside the catalytic cleft. (**A**) Representative structure of a kinase, exemplified by the MAPK p38 and illustrating the N- (green) and C-lobe (grey), as well as the activation loop and ATP binding site (PDB: 2Y8O). (**B**) Structural examples for docking interactions. Peptides are depicted in yellow, with key residues highlighted, and mapped on the surface of N- (green) or C-lobe (grey) of the various kinases (PDBs: 2OKR (p38); 5ODT (Aurora A); 3BEG (SRF1); 5VLP (PDK1). (**C**) Axin peptide (yellow) binds to the GSK3β kinase docking pocket and acts to recruit substrate proteins (PDB: 1O9U). (**D**) Domain representation of PLK1 and structure of PLK1 PBD bound to the phosphorylated peptide of the PLK1 substrate PBIP1 (Polo-box-interacting protein 1) (PDB: 3P37). (**E**) Structure of CDK2 bound to cyclin A2 which interacts with a substrate peptide of cyclin-dependent kinase inhibitor p27 (PDB: 1H27).

More than 500 human kinases catalyse the transfer of a phosphoryl group from ATP onto a target protein. Kinases are broadly categorised in serine/threonine and tyrosine kinases, with serine/threonine kinases representing the majority. Kinases can be divided into seven groups: the **AGC** (PKA, PKG and PKC family of kinases), **CAMK** (calcium/calmodulin-dependent protein kinases), **CK1** (casein kinase 1), **CMGC** (cyclin-dependent kinases (**C**DKs); mitogen-activated protein kinases (**M**APKs); glycogen synthase kinase 3 (**G**SK3); CDK2 like kinases (**C**LKs)), **STE** (homologues of yeast Sterile 7, Sterile 11, and Sterile 20), **TK** (tyrosine kinases), **TKL** (tyrosine kinase-like kinases) and **atypical** group of kinases [15]. Most kinases share a common structure, with an N-terminal lobe that binds to ATP and a C-terminal lobe that binds to the substrate (Figure 2A). The specificity for the residue to be phosphorylated is determined by the depth of the catalytic cleft, and the specificity for the region is determined by the residues flanking the site of the catalytic activity [16]. Kinase specificity and catalytic activity can be increased by establishing supplementary interactions that tether the kinase to the substrate [17]. These interactions can be mediated either by docking to the kinase domain itself or by binding to auxiliary modular domains [16,18] (Figures 1B–D and 2). As a variation, cyclins act as distinct protein moieties to modulate the activity of cyclin-CDKs and to offer substrate binding sites [19]. In the following section, we survey the different kinds of docking interactions, in which serine/threonine kinases are reported to engage.

### Docking motifs of MAPKs

MAPKs play a central role in the transfer of extracellular signals to intracellular responses [20] and phosphorylate a wide range of substrates, with distinct specificities exhibited by different family members. Docking interactions are commonly employed by MAPKs [18,21]. Briefly, the MAPKs harbour an acidic common docking site outside of the catalytic site, which forms contacts with the kinase interacting motif on MAPK substrates [22,23]. The basic amino acid stretch of the docking motif is followed at varying linker length by a hydrophobic amino acid stretch. Together, this reconstitutes the classical basic-hydrophobic motif bound by the MAPKs [24], as exemplified here by the MAPKAP kinase 2 (MKK2) peptide (_370_-IKIKKIEDASNPLLLKRRKKARAL-_393_) bound to p38 [25] (Figure 2B). MAPKs have also additional binding sites for docking motifs, such as the F-site of MAPK1 (ERK2) that binds to peptides with the consensus motif Fx[F/Y]P [26–28]. More complex recognition mechanisms may be in place in order to allow for co-regulation of substrates by different MAPKs, as exemplified by conformation-dependent binding of the activating transcription factor 2 (ATF2) to either p38 or the Jun N-terminal kinase 1 (JNK1) [29]. Several studies have been aimed at systematically defining and identifying MAPK docking motifs [24,30]. In a recent study, candidate docking motifs in the human proteome were screened for interaction with the MAPKs p38 and JNK1 in a yeast-based approach. The screen confirmed known motif-containing interactors and unravelled non-classical docking motifs [30].

Underlining the central role that docking interactions play in orchestrating MAPK activity, docking motifs can be found in the upstream MAPK kinases (MKKs) and downstream MAPK phosphatase (MKPs) modulators of MAPK signalling. In the case of the MKKs, the interaction paradigm is flipped as the kinase (the MKK) harbours the motif and not the substrate (the MAPK). Examples include the binding of the JNK kinases to a docking motif in MKK4 and MKK7 [31,32], ERK1/2 binding to MKK1/2 [33], as well as the docking site in MKP5 for p38 [34]. Together, this creates the notion of different MAPK interactors (kinases, phosphatase and substrates) essentially competing for MAPK binding and hence fine-tuning MAPK signalling [35,36]. Lastly, docking site interaction can also be subjected to phospho-regulation, as exemplified by the phosphorylation of the p38 docking groove at T123 which impacts p38 ability to bind and phosphorylate its substrates [37].

### Docking motifs of the PI3K-related (PIKK) family of kinases

Docking motifs have also been reported to be involved in the substrate targeting of the phosphatidylinositol-3 kinases-related (PIKK) family of atypical kinases that are involved in several stress responses, including DNA damage, errors in mRNA splicing and availability of nutrients [38]. The PIKK kinase family is characterised by N-terminal helical HEAT repeats, an intermediate FAT domain and a C-terminal kinase domain [39]. The N-terminal HEAT repeats have been reported to engage in docking interactions. For instance, the kinase ATM (ataxia telangiectasia mutated) is recruited to DNA double-stranded breaks by a C-terminal stretch in nibrin (NBN; _734_-AK**EE**SLA**DD**LF**RY**NPYLKRRR-_754_) [40]. Similarly, the related kinases ATR (ataxia telangiectasia and rad3-related protein) and DNA-PKcs (DNA-dependent protein kinase, catalytic subunit) are also targeted to substrates by interactions with acidic C-terminal stretches [40–42].

In contrast, the mechanistic target of rapamycin (mTOR), another member of the PIKK family, uses an auxiliary domain for substrate recognition. The mTOR complex 1 (mTORC1) is centrally controlling cellular growth by integrating extra- and intracellular signalling to coordinate synthesis and degradation of essential biomolecules, such as nucleic acids, lipids and amino acids [43]. mTOR is a component of the mTORC1, which contains, among other auxiliary proteins, the regulatory-associated protein of mTOR (raptor) [43]. As regulatory subunit, raptor directs mTOR activity by binding to a TOR signalling (TOS) motif (FxΦDΦ; where Φ is a hydrophobic amino acid) found for example in the eukaryotic initiation factor 4E binding proteins (4E-BP) 1, 2 and 3 and ribosomal S6 kinases (RSKs) [44–46]. Adding to the complexity, 4E-BP1 contains in addition to the C-terminal TOS motif, a N-terminal RAIP motif which binds a discrete site on the raptor and is required for the sequential phosphorylation of 4E-BP1 by mTORC1 [47,48]. Furthermore, the FKBP12-rapamycin-binding domain on mTOR itself has been suggested to provide a secondary substrate-recruitment site, which is contacted by RSKs and mTORC1 inhibitors [46,49].

### Examples of docking motifs binding to individual kinases

Several docking motifs have been proposed for specific kinases rather than kinase families, as exemplified here with the docking motifs of the aurora A kinase, the SR protein kinase 1 (SRPK1) and the phosphoinositide-dependent kinase-1 (PDK1) (Figure 2B).

The aurora A kinase is centrally involved in cell cycle regulation. It binds to hydrophobic docking motifs. One of its targets (targeting protein for Xklp2; TPX2) was shown to bind the kinase using two distinct hydrophobics motifs, with a _7-_**Y**S**Y**D**A**PSD**F**IN**F**SS_-21_ peptide interacting with the N-lobe and a _30-_NIDS**WF**EEKANLEN_-43_ stretch being accommodated between the N- and the C-lobe [50]. A hydrophobic aurora A docking motif was also found in the transforming acidic coiled-coil-containing protein 3 (TACC3; _522-_EES**F**RDPAEVLGTGA_-536_) with F525 being a critical determinant of binding (Figure 2B). Aurora A-mediated phosphorylation of TACC3 (on S558) induces the transition of an intrinsically disordered region to an α-helical state, which facilitates the concomitant binding of clathrin to yet another binding motif in TACC3 [51]. Thus, the effects of phosphorylation on SLiM binding, which will be discussed later, may be both direct and indirect.

The kinase SPRK1 is involved in the regulation of splicing. It binds to a docking motif (_201-_SYGRSRSRSR-_210_) in its substrate the serine–arginine protein ASF/SF2 (Figure 2B). Kinase targeting further requires an additional ASF/SF2 peptide (_191-_RVKVDGPR_-198_), which controls successive ASF/SF2 phosphorylation [52]. Proposedly, ASF/SF2 is first threaded through the docking site of the kinase for subsequent phosphorylation, allowing for the _191-_RVKVDGPR_-198_ peptide to bind to the docking groove before _201-_SYGRSRSRSR-_210_ [53].

Finally, kinase docking interactions may themselves be phospho-dependent. For example, the phosphoinositide-dependent kinase PDK1 is crucial for activating several members of the AGC family of kinases. PDK1 is targeted to the AGC kinases by interactions with a hydrophobic PDK1-interacting fragment (PIF) docking motif in the C-terminal lobe of the AGCs kinase domain [54–57]. The binding of the PIF motif by PDK1 is in turn regulated by phosphorylation of the S/T residue in the motif (Fxx[F/W/Y][pS/pT][F/Y]; Figure 2B).

### GSK-3β and docking interactions with the scaffold protein axin

Docking interactions can be made between kinases and scaffold proteins rather than directly with the substrate, as shown for the glycogen synthase kinase 3 β (GSK-3β), which serves as a negatively regulator in the control of glucose homeostasis. GSK-3β binds a 19 amino acid peptide (_383_-VEPQKFAEELIHRLEAV-_399_) in axin, which folds into a single amphipathic helix accommodated in a hydrophobic binding pocket (Figure 2C). Axin acts as a scaffolding protein in the β-catenin destruction complex, and brings together GSK3β and β-catenin, which enhances β-catenin phosphorylation and subsequent degradation [58]. Recently, it was shown that the enhancement of the catalytic rate by the scaffolding interaction was fairly modest, but that the axin-mediated scaffolding increases the specificity of GSK3β for β-catenin in a cellular setting with many competing substrates [59]. The docking interactions between GSK-3β and axin thus follow a similar principle for specificity increase as the docking interactions between the protein phosphatase 1 (PP1) and its different regulatory subunits discussed later.

### Docking interactions of kinases with auxiliary SLiM-binding domains

Docking interactions may be mediated by auxiliary protein domains that are covalently linked to the kinase domain. This is frequently observed for tyrosine kinases (e.g. using SH2 and SH3 domains for targeting) [16] but more rarely for serine/threonine kinases. However, there are some exceptions including the polo-box domain (PBD) in polo-like kinases (PLKs), the CCT domain found in the ste20-related proline alanine-rich kinase (SPAK) and oxidative stress response kinase (OXSR1).

PLKs have critical roles in regulating cell cycle progression. Among the PLK family members, PLK1 and PLK4 have been most studied and they have well-established function as mitotic regulators [60,61]. PLKs are characterised by the presence of a N-terminal kinase domain and a C-terminal PBD composed of two polo boxes (Figure 2D), except for PLK4 which has a second cryptic PBD. The PBD is critical for the subcellular localisation of the kinases [62] and mediates substrate targeting by engaging in phospho-dependent interactions with docking motifs [63]. The consensus motif for the PLK1 PBD was shown to be [H/N/M/T]S(pT/pS)[P/x] based on peptide library screening [63,64]. Crystal structures between a MGS(pT)PL peptide and the PLK1 PBD revealed that both polo boxes formed contacts with the phospho-peptide [64,65] (Figure 2D). Various PBDs recognise similar sequences [64,65]. However, the PBD confer specificity among the PLK family suggesting little redundancy in their functionality [66]. A recent report inferred the contribution of an additional tyrosine-rich pocket close to the phospho-peptide binding pocket to their substrate specificity [67].

The closely related kinase SPAK and OXSR1 are also targeted to their substrates by docking interactions mediated by an auxiliary domain, in this case, a CTT domain. The CTT domain of SPAK and OXSR1 binds to a RFx[I/V] motif found in the two substrates kinases WNK1 and WNK4 [68,69]. A proteomic peptide phage display screen against the OXSR1 CCT domain recently confirmed the RFx[I/V] motif in WNK1 and WNK4 and suggested additional ligands that may serve as substrates for the kinase, including the protein dedicator of cytokinesis protein 1 (DOCK1) [70].

### Docking motifs of cyclins

The CDKs need a separate subunit, called cyclins for targeting substrates to the catalytic units. Due to their cell cycle stage-dependent accumulation and degradation, cyclins emerge as key for controlling cell cycle progression [19,71]. The interactions between substrates and cyclins involve SLiM-based interactions. Several cyclins recognise a classical RxL docking motif and accommodate the motif in a hydrophobic pocket, as first identified in the context of the cyclinA–CDK2-p27 complex [19,72] (Figure 2E). Cyclins have been found to bind to variations of the RxL motif, or to alternative motifs, including the LxF motif (CLB2; mitotic cyclin in budding yeast) [73], the PxxPxF motif (CLB3; G2 cyclin in budding yeast) [74], the NLxxxL motif (CLB5/6; S-phase cyclin in budding yeast) [75] and a leucine and proline-rich motif (late G1 CDKs in budding yeast) [76]. The latter was recently demonstrated to tolerate substantial residue variation at position p + 4 and p + 6 of the PxxL motif, which fine-tuned the affinity and resulted in time-dependent substrate phosphorylation in budding yeast [77]. The docking interactions between cyclins and the substrates may further involve a combination of SLiM-binding sites as shown for the binding of the retinoblastoma protein Rb to cyclin D. The interaction between the two proteins involves distinct SLiMs, including a classical cyclin RxL docking motif that dock the hydrophobic pocket of the cyclin, a LxCxE motif found in cyclin D that binds to a docking groove on Rb, and a C-terminal helix of Rb that docks to cyclin D [78,79].

## The readers of the phosphorylation code

As described, targeting of serine/threonine kinases to their substrates does at least for a subset of enzymes involve SLiM-based interactions. This contributes to determining kinase substrate specificity in a time- and space-dependent manner. The enzymes phosphorylate in turn sites in the intrinsically disordered regions of their target proteins, potentially affecting the binding properties of their SLiMs in different ways. Phosphorylation of SLiMs may turn on interactions with obligate phospho-binders, which are commonly referred to as readers of the phosphorylation code (Figure 1C). Alternatively, phosphorylation of residues interspersed or in the flanking regions of motifs may act to fine-tune affinities. In this review, we refer to the latter as phospho-modulation and discriminate it from the obligate phospho-binding of domains.

### Obligate phospho-binders

**Figure 3.**
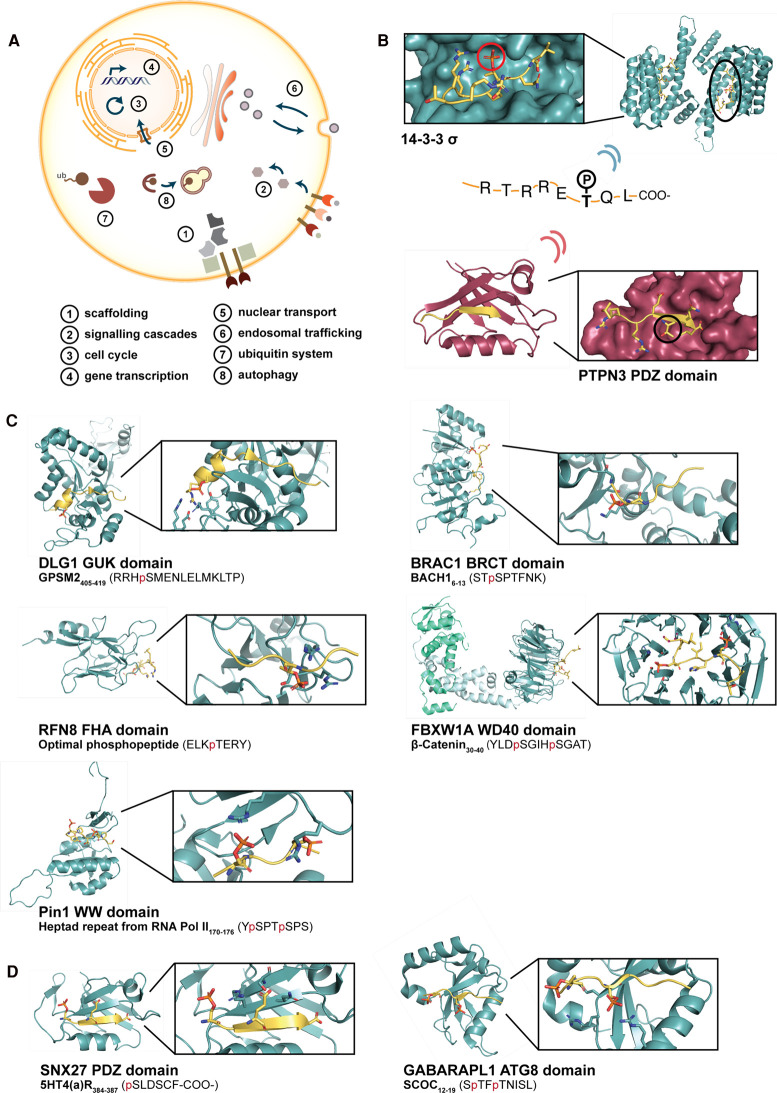
Overview of a select set of phospho-regulated SLiM-based interactions. (**A**) Overview of the cellular functions governed by phosphorylation. (**B**) Phospho-switch mediated by T158 phosphorylation on the HPV16 E6 oncoprotein binding either 14-3-3 domains (phosphorylated, PDB: 6WTZ) or PDZ domains (unphosphorylated, PDB: 6HKS). (**C**) Representative structures of obligate pS/pT-binding domains (cyan) and phosphorylated peptides (yellow, phosphate group in red). Interacting residues on the domain are highlighted (PDBs: 3UAT (DLG1 GUK domain), 1T15 (BRCA1 BRCT domain), 2PIE (RFN8 FHA domain), 1P22 (FBXW1A WD40 domain), 1F8A (PIN1 WW domain). (**D**) Representative structures of peptide-binding domains, the binding of which is subjected to phospho-modulation in the vicinity of the core binding motif. Peptides are depicted in yellow and interacting residues on the domains are highlighted (PDBs:) 7AA9 (GABARAPL1 ATG8 protein), 5EM9 (SNX27 PDZ domain).

The obligate phospho-binders described here require a phosphorylated serine or threonine for binding. They include for example the 14-3-3 proteins, the forkhead-associated (FHA) domains, the BRCA1 C-terminal (BRCT) domains, the peptidyl-prolyl isomerase 1 (PIN1) WW domain, and several WD40 repeat proteins [80,81]. These domains have crucial roles in governing cell function, including cell cycle progression and DNA damage response [80]. Another obligate phospho-binder is the kinase-dead guanylate kinase (GUK) domain, which has a scaffolding role at the postsynaptic density [82]. A common mechanism of pS/pT recognition of these phospho-binding domains is contacting the phosphate group either by positively charged amino acids (H/K/R) or by hydrogen bonding, including the peptide backbone or residues with free hydroxyl groups (for example S/T). In the following section, we provide some representative cases of the interactions of the readers of the phosphorylation code and relate the interactions to cellular functions (Figure 3A).

#### 14-3-3 Adaptor proteins bind to pS/T containing SLiMs

The 14-3-3 proteins represent the first discovered, and likely most well-studied, pS/T binding domains. The seven 14-3-3 isoforms (β, ɛ, η, γ, τ, ζ and σ) commonly form hetero- and homodimers [83]. The proteins adopt a W-shaped fold, with the monomers arranging in α-helices and each providing a positively charged phospho-peptide binding pocket [83,84]. The 14-3-3 proteins bind similar ligands but with varying affinities [85]. A canonical binding motif has been defined (Rxx[pS/pT]xP), where both pS and pT are accepted [86] (Figure 3B). The 14-3-3 binding motif matches well with motifs modified by basophilic kinases (AGC and CAMKs) but with a unique preference for a proline at position p + 2 [87]. However, variants of the 14-3-3 binding motif have been described [88], and the proline is absent in many known 14-3-3 ligands [87]. There are also sporadic reports on phospho-independent 14-3-3 interactions, in which an acid residue mimics the phosphorylated residue [70,89–91].

Due to their abundance and large number interaction partners, 14-3-3 proteins have been implicated in numerous cellular processes, such as signal transduction, cell cycle progression, apoptosis and metabolism [83,84,92] (Figure 3A). For instance, a recent study established the interaction between 14-3-3 adaptor proteins and phosphorylated cardiac membrane protein phospholamban, protecting the latter from dephosphorylation and possibly contributing to prolonged muscle contractility [93]. The 14-3-3 proteins emerge further as key regulators of kinase activity by allosteric mechanisms [94]. As an example, 14-3-3 adaptor proteins have been shown to retain the calcium/calmodulin-dependent protein kinase kinase-2 (CAMKK2) in an inactivated state by binding to pS100 and pS511 on the kinase [95]. Furthermore, the constitutive dimerisation of the 14-3-3 proteins has been demonstrated a molecular tool for the regulation of kinase activity. For example, the serine/threonine-protein kinase B-raf forms an asymmetric dimer, held together by the 14-3-3 adaptor proteins, in which one kinase is active and one is inactive [96]. The formation of a heterodimer between MKK2 and B-raf is also promoted by bridging of the 14-3-3 proteins in both the active and inactive state of the kinase dimer [97].

#### MAGUK kinase-dead GUK domains have evolved to recognise pS/pT

Among the less recognised phospho-binding domains are the kinase-dead GUK domains of MAGUKs (membrane-associated GUKs). MAGUKs are scaffolding proteins, which are abundantly present at cell–cell junctions and pivotal for biological processes such as cell polarity, intercellular communication and cell adhesion [82] (Figure 3A). They are characterised by the presence of a signature GUK domain, which has lost its enzymatic activity but engages in phospho-dependent SLiM-based interactions [82]. Residues from the former GMP binding site contact the phosphorylated moiety, as shown for the binding of a phosphorylated peptide the GUK domain from DLG1 (discs large homologue 1) [98] (Figure 3C). Phospho-dependent interactions have also been found for the GUK domains of several other MAGUKs [82]. For example the PSD95 (postsynaptic density protein-95) GUK domain was found to bind to the DLGAP (disks large-associated protein) in a phospho-dependent manner, and the interaction was shown to be required for the maturation of postsynaptic densities and synaptogenesis [99].

#### BRCT domains bind pS-containing SLiMs

Some phospho-binding domains distinguish between pS and pT. For example, the BRCT domain specifically bind pS-containing SLiMs. The domain was, as the name suggests, originally identified in the tumour suppressor gene BRCA1 (breast cancer type 1 susceptibility protein). The BRCA1 BRCT domain was found to bind to the S990 phosphorylated transcription regulator protein BACH1 in the context of the pSxxF motif [100,101] (Figure 3C). The observation that primary contacts to the phospho-peptide are made by S1655 and K1702 is considered the paradigm for BRCT-mediated phospho-recognition. Nonetheless, non-canonical phospho-peptide recognition by a conserved basic surface has been reported for the interaction of phosphorylated keratin (CYK4) with the BRCT domain of the protein ECT2 [102]. Numerous other phospho-binding BRCT domains are found in proteins involved in DNA replication and repair, and in cell cycle checkpoint maintenance and cytokinesis [80,102–105]. Recently, a modelling approach identified phospho-peptides from DNA endonuclease RBBP8 (pS347) to bind to the FHA-BRCT1/2 domains from nibrin, a protein involved in processing double-stranded DNA breaks [106]. Furthermore, some BRCT containing proteins harbour more than one BRCT domain, with several domains contributing to substrate specificity [107]. Finally, BRCT domains have also been reported to engage in phospho-independent interactions [108]. As an example, the BRCT domains from BRCA1-associated RING domain protein 1 (BARD1) were shown to interact with lysine-ubiquitinated histone H2A which resulted in its and concomitant BRCA1 recruitment to sites of double-stranded DNA breaks [109].

#### FHA domains bind pT-containing SLiMs

The FHA domains represent a domain family that recognises phospho-peptides with a strict requirement for pT [110,111]. The FHA domain-containing proteins are implicated in various cellular functions such as DNA damage response, cell cycle regulation and signal transduction [80,112] (Figure 3A). The domain is composed of about 100–120 residues that fold into a 11-stranded β-sandwich. Five conserved residues define two pockets that recognise a conserved pT, with two different binding motifs reported in the ELM database (pTxx[I/L/V]; pTxx[E/D]) [88,111,112] (Figure 3C). Based on structural analysis, it was found that additional hydrogen bonds and accommodation of the methyl group from threonine in a hydrophobic pocket are responsible for the preference of the FHA domain for pT over pS [111,113]. Numerous examples for the pT-dependent interactions of FHA domains have been reported [80,112]. For instance, the FHA domain of MDC1 (mediator of DNA damage checkpoint protein 1) was found responsible for the recruitment of phosphorylated USP15 to double-stranded DNA breaks [114]. Another recent example includes the recruitment of the E3 ubiquitin ligase pellino1 via its FHA domain to DNA damage sites by binding to T18 phosphorylated p53 [115]. Additional recognition modes such as the binding of the FHA domain of PNKP (polynucleotide kinase 3′ phosphatase) to the pS–pT di-phospho-peptide on the DNA repair protein XRCC1 have been elucidated [116]. Lastly, FHA domains have also been reported to also mediate protein dimerisation and oligomerisation [95], as exemplified by T9-phosphorylation induced oligomerisation of the protein TIFA (TRAF-interacting protein with an FHA domain) [117].

#### WD40 domains and the paradigm of phosphodegron recognition

WD40 repeats are β-propeller domains that constitute one of the largest domain families in the human proteome [118]. Several WD40 interactions have been found to be phospho-dependent, including binding of degradation motifs (or degrons) that promote the interactions with E3 ubiquitin ligases and consequently ubiquitin conjugation [119,120] (Figure 3A). The paradigm of phospho-dependent WD40 degron recognition involves the F-box proteins, which act as adaptor proteins in the Skp, Cullin, F-box containing ubiquitin complex [121,122] The complex is involved in the degradative processes during the cell cycle and substrate recognition [119,123]. In early studies, it was shown that the WD40 domains of the F-box protein F-box/WD repeat-containing protein 1A (FBXW1A) and 7 (FBXW7) engage in phosphodegron interactions with their double phosphorylated substrates β-catenin (_30_-YLDpSGIHpSGAT-_86_) and cyclin E (_374_-LPSGLLpTPPQpSG-_385_), respectively. Both phospho-peptides pack against the narrow side of the channel formed by the β-propeller of the WD40 domains [124,125] (Figure 3C). More recently, it was shown that the turn-over of transcription factors such as the krueppel factor 10 (KLF10) is regulated by phosphorylation (_8_-(pT)PPY(pS)P-_86_) creating a phosphodegron recognised by FBXW7 [126]. Notably, the phosphodegrons recognised by FBXW7 are frequently mediated by GSK-3β, that phosphorylates sites four residues upstream of an already phosphorylated residue to create the double phosphorylated ligand [127,128]. In addition, FBXW1A has been reported to be responsible for phospho-dependent interaction with and hence degradation of, for example, the wee1-like protein kinase (WEE1; pS53, pS123) [129], the M-phase inducer phosphatase 2 (CDC25B; pS101, pS103) [130], and the eukaryotic elongation factor 2 kinase (EEF2K; pS499) [131].

There is thus a frequent cross-talk between phosphorylation and degradation as illustrated by the interactions of the WD40 domains of FBXW1A and FBXW7 that both serve as substrate recognition components of E3 ligase complexes.

#### The PIN1 WW domain is an obligate phospho-binder, but not the rest of the WW domain family

PIN1 is a peptidyl-prolyl cis–trans isomerase that specifically isomerises a phosphorylated p[S/T]P motif and that has essential roles in regulating cell cycle progression [132,133]. It has a WW domain that engages in phospho-dependent interactions with targets of PIN1 isomerase activity. For example, phosphorylation of two conserved threonine residues in the M-phase inducer phosphatase 3 (CDC25C) is required for its binding to the PIN1 WW domain [134]. The structural requirements for the recognition of the pS/pT-P containing peptides have been exemplified using a phospho-peptide derived from the C-terminal domain of the RNA polymerase II [135] (Figure 3C). The pS-P recognition motif of PIN1 overlaps with sequences modified by CDKs, which is of relevance for PIN1's role in cell cycle progression. For example, the oncogenic transcription factor myb-related protein B (MYBL2) is phosphorylated by CDKs, which creates a PIN1 WW domain binding motif and results in PIN1-mediated conformational changes. As a consequence, sites in MYBL2 become available for further phosphorylation allowing it to assume its regulatory role in gene transcription in a hierarchical manner [136]. An unusual interaction sequence was reported for the binding of CPEB1 (cytoplasmic polyadenylation element-binding protein) to PIN1 WW domain that occurs prior to, but is enhanced by, phosphorylation. The conformational change induced on CPEB1 by PIN1 results in its subsequent ubiquitination and degradation [137,138]. In contrast, PIN1-mediated isomerisation of the bromodomain-containing protein 4 (BRD4) protects the protein from degradation, which was found to be dependent on PIN1 WW domain binding to the T204 phosphorylated substrate [139].

Importantly, there are about 100 human WW domains that are involved in a variety of cellular processes including receptor signalling, protein trafficking and degradation, and RNA transcription and processing [140,141]. The remainder of the WW family binds proline-rich peptides without requirement for phosphorylation, with the main motif classes being [L/P]PxY and PPPL [142,143]. Even though not compulsory for the interaction, WW domain-mediated binding can be tuned by phosphorylation. For example, binding of the E3 ligase NEDD4's WW domains was enhanced by phosphorylation on a serine preceding the PPxY motif found in the C-terminus of connexin43 [144]. A complex interplay between the SMAD family members 1-5 phosphorylation and a set of ‘reader’ WW domains has been proposed to mediate transforming growth factor β (TGFBβ) and bone morphogenic protein (BMP) signalling downstream of the receptor. Sequential phosphorylation allows for the binding of WW domains first of proteins involved in SMAD activation and then subsequently of proteins involved in its degradation [145]. In a follow-up study, it was demonstrated that the interaction of the central regulatory SMAD7 with SMAD1-5 was independent of this phospho-code [146].

### Many SLiM-based interactions are modulated by phosphorylation

In addition to the obligate phospho-binders, there is a vast amount of protein domains for which the binding does not critically rely upon phosphorylation, but for which there is a fine-tuning of affinities. Phosphorylation can occur within the motif or in its flanking regions, with varying effects on binding (Figure 1C). Further, phosphorylation may block binding of one modular domain, and at the same time favour binding of another binder thus resulting in interaction partner switching [3] (Figures 1A and 3B). Due to the ubiquity of phosphorylation as a regulatory mechanism, a comprehensive annotation exceeds the scope of this review. Instead, we exemplify common themes by focussing on selected cases among binding domains involved in scaffolding interactions or protein trafficking processes.

#### PDZ domain-mediated interactions

PSD-95, Disks large tumour suppressor and Zonula occludens 1 (PDZ) domains represent one of the largest domain families with over 260 PDZ domains found in over human 150 proteins. PDZ proteins are ubiquitous scaffolding modules involved, among many processes, in the organisation of signalling complexes at membranes, and in the trafficking of receptors [147] (Figure 3A). The canonical PDZ binding motifs are divided into three main classes: class I a [S/T]xɸ-coo-, class II ɸxɸ-coo- and class III [E/D]xɸ-coo- found at the C-terminus of its interaction partners with various specificity classes [147]. Class I PDZ binding motifs harbour a S/T residue at the p − 2 position amenable to phosphorylation (with C-terminal residue indicated as p0). Phosphorylation at this position typically abrogates PDZ binding (Figure 3B) [148,149]. However, there are exceptions from the rule, such as the binding of the Na^+^/H^+^ exchange regulatory cofactor NHE-RF1 (NHERF1) PDZ domain to the C-terminus of the β2-adrenergic receptor, which is enabled by serine phosphorylation at the p − 2 position [150]. Another example of PDZ binding being tuned by phosphorylation of motif-flanking residues is the binding of sorting nexin-27 (SNX27) PDZ domain. In this case, phosphorylation at the p − 3 and p − 5 positions enhances binding, whereas at p − 2 abrogates binding and likely impacts transport by the SNX27–retromer complex [148] (Figure 3D). Such ligand phosphorylation events may also contribute to regulating specificities among the large number of PDZ domain proteins with the potential to bind class I ligands [151]. Along these lines, the effects of phosphorylation at the p − 3 position of the class I PDZ binding motif found in the C-terminus of the RSK on PDZ domain binding was systematically investigated. Using a high-throughput hold-up assay against a collection of almost all human PDZ domains it was found that the p − 3 phosphorylation dramatically altered the PDZ domain binding landscape of RSK1 [152]. As a variation, PDZ switching following phosphorylation has been proposed to play a role in dendrite development by orchestrating the association of delta-catenin with either of the PDZ proteins MAG1 (membrane-associated guanylate kinase inverted 1) or PDLIM5 (PDZ and Lim domain protein 5) [153].

Beyond mediating switching between PDZ domains, it was shown that p − 2 phosphorylation may simultaneously block PDZ binding while creating a 14-3-3 binding site, leading to switching between distinct types of binding domains (Figure 3B). Along these lines, the C-terminus of the HPV16 E6 oncoprotein contains an overlapping PDZ/14-3-3 binding site. Phosphorylation of the motif attenuates PDZ domain binding while concomitantly recruiting 14-3-3 proteins [154,155] (Figure 3B). The concept of 14-3-3/PDZ switching has been extended to several other proteins that similarly harbour overlapping binding motifs [156]. In further support, phosphorylation of the p − 1 and p − 2 position in the PDZ binding motifs of RSK was shown to promote 14-3-3 binding [149]. PDZ/14-3-3 interactor phospho-switching may thus be a common and biological regulatory paradigm. Overall it becomes evident that phosphorylation acts as a key mechanism to control and fine-tune PDZ domain interactions.

#### VHS domains and targeting of cargo for endocytosis

Phosphorylation regulates the binding of the endocytic transport signals (Figure 3A). For example, the GGA (Golgi-localised, γ-ear-containing, ADP-ribosylation-factor-binding)-mediated sorting into the endosome has been proposed to be regulated by phosphorylation. The GGA proteins (GGA1-3) share a VHS (Vsp27p/Hrs/STAM) domain that binds to DxxLL containing sequences. It was shown that the association of the GGA3 VHS was enhanced by serine phosphorylation at the p − 1 position relative to the core DxxLL motif in the cation-independent mannose 6-phosphate receptor (IGF2R) [157]. Similarly, serine phosphorylation at the p + 3 position in the DxxLL motif of β-secretase (BACE2) led to an increase in affinity for the VHS domains of the GGA proteins [158].

#### ATG8 proteins and targeting proteins for autophagy

During autophagy, cytoplasmic components are engulfed by autophagosomes. The autophagy-related protein 8 (ATG8) proteins are central components of the autophagosome forming machinery and can act as membrane scaffolds and cargo receptors [159]. The ATG8 proteins include the microtubule-associated proteins 1A/1B light chain 3 (MAP1LC3A,-B,-C) and gamma-aminobutyric acid receptor-associated proteins (GABARAP,-L1,-L2). The ATG8 proteins interact with selective autophagy receptors, which in turn bind ubiquitinated proteins targeted for degradation [160] (Figure 3A). The proteins recognise LC3-interacting region (LIR) motifs with a general consensus [F/Y/W]xxɸ, which was originally identified in sequestosome 1 (_334_-GDDD**WTHL**SSK-_344_) and later found in a large number of other proteins [70,160–162].

The LC3–LIR interactions have been shown to be augmented by ligand phosphorylation at different positions. Several studies have reported that phosphorylation of the p − 1 position upstream of the motif enhances the affinity of the interactions [163–166]. Phosphorylation at sites more upstream the LIR motif may also increase the affinity of the interactions as shown for the LIR motif found in beclin-1 (p − 1, p − 3 and p − 7) and in PIK3C3 (phosphatidylinositol 3-kinase catalytic subunit type 3) (p − 1, p − 6) [167]. Additionally, it has been shown that the LIR motif of SCOC (short coiled-coil protein), a positive regulator of starvation-induced autophagy, is positively regulated by phosphorylation of positions both downstream and upstream of the LIR motif (p − 2, p + 2 and p + 5) [168] (Figure 3D). Similarly, a synergistic effect was observed between p − 1 and p + 6 phosphorylation in the flanking regions of a dormant LIR motif in integrin β3 that transformed weak interactions to low micromolar affinities [169]. Together these examples suggest that multiple residues in close vicinity of the LIR motif are subjected to phosphorylation to fine-tune ATG8 domain binding and thereby regulate the targeting of proteins for autophagy.

#### Karyopherins binds nuclear localisation signals for nuclear import

Along the lines of regulation of targeting motifs by phosphorylation, there are also ample reports on how nuclear localisation is phospho-regulated (Figure 3A). Karyopherin α (or importin α) family members bind to nuclear localisation signals (NLSs) and transports cargo through the nuclear pore complex [170]. They have two NLS binding pockets and bind basic bipartite NLSs that are about 17–19 amino acids long. They also bind basic monopartite NLSs that have been divided into several different classes, of which some bind to either the major or minor pocket, and others interact with both pockets [70,171]. The function of NLSs can be up or down-regulated by phosphorylation upstream or downstream the motifs [172]. For instance, pS350 phosphorylation upstream of the NLS was reported to attenuate nuclear shuttling of DAPK2 (death-associated protein kinase-related apoptosis-inducing protein kinase 2; _346_-DSSMVpS**KR**F**R**F-_356_) [173]. In contrast, phosphorylation of S360 located downstream of the NLS (_354_-AR**KRK**P**S**P-_361_) in IRF2BP2 (interferon regulatory factor 2 binding protein 2) was found to be necessary for its nuclear localisation [174]. No specific karyopherin has been reported for the here described phospho-modulated NLS, thus, there are no structural details of the interactions.

Taken together, the tuning of affinities of SLiM-based interactions may be an underappreciated aspect of the functional consequences of phosphorylation due to the large number of proteins engaging in phospho-modulated binding events.

## The erasers — docking interactions of phosphatases

**Figure 4.**
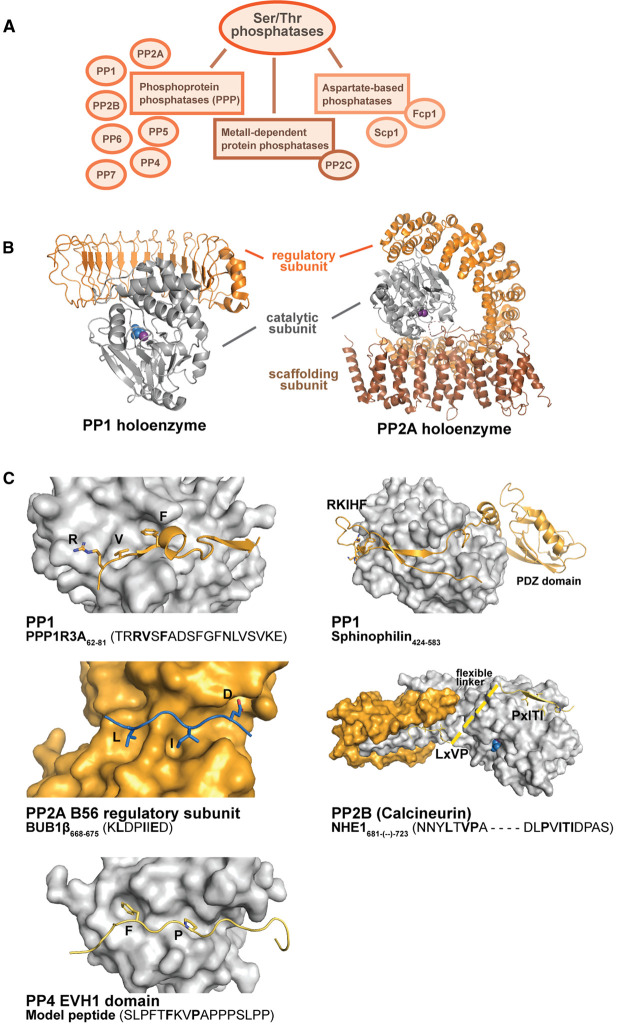
Strategies of serine/threonine phosphatases for substrate targeting. (**A**) Overview of the serine/threonine phosphatase classification. (**B**) Representative holoenzymes of PP1 (PDB: 6OBP) and PP2A (PDB: 2NPP). The catalytic subunit is depicted in grey, whereas the regulatory subunit is shown in orange and the scaffolding subunit of PP2A in brown. (**C**) Docking interactions engaged by the different PPPs with either peptides of regulatory subunits (orange) or substrates (yellow). An exception is the substrate peptide from BUB1β depicted for clarity in blue on the orange regulatory subunit B56. Key residues on the peptides are highlighted (PDBs: 5ZQV (PP1 with PPP1R3A), 3EGG (PP1 with sphinophilin), 5JJA (B56 PP1 regulatory subunit), 6NUC (PP2B), 6R8I (PP4)).

Finally, we focus on the SLiM-based docking interactions of the protein phosphatases that counteract the actions of kinases and thereby contribute to controlling the specificity and length of phosphorylation events. In contrast with the common structure of the kinases (Figure 2A), phosphatases have more diverse structures and different mechanisms of hydrolysis [175]. The human serine/threonine phosphatases are encoded by about 40 genes [176]. There are three superfamilies of serine/threonine phosphatases: phosphoprotein phosphatases (PPPs; PP1, PP2A, PP2B (calcineurin), PP4–PP7), metal-dependent protein phosphatases (PPMs; PP2C) and the aspartate-based phosphatases [176] (Figure 4A). Here, we focus on the PPPs. PPPs rarely exist as free catalytic subunits, but form holoenzymes together with regulatory subunits, resulting in a combinatorial assembly of phosphatase complexes. For example, more than 200 regulatory subunits have been found for PP1 and over a dozen regulatory subunits and isoforms have been found for PP2A [177]. All PPPs share a common catalytic core and catalytic mechanism. Variations in the solvent-exposed residues surrounding the catalytic site provide some specificity for the dephosphorylation reaction, but there is so far no reliable consensus motifs for the sites of PPPs dephosphorylation [177]. Docking interactions contribute to the substrate specificities, as exemplified below by PP1, PP2A, calcineurin and PP4. For other phosphatases, the potential docking motifs still remain to be established [177].

In addition to the serine/threonine phosphatases discussed here, there are also dual-specificity phosphatases (DUSPs), which may also use motifs for substrate targeting as recently convincingly outlined for yeast CDC14 [178]. The phosphatase was further demonstrated to specifically dephosphorylate pSP-containing peptides [179]. We consider diving into further mechanistic details of the DUSPs as beyond the scope of this review.

### PP1

PP1 is responsible for catalysing a large proportion of eukaryotic protein dephosphorylation events [180]. There are three PP1 genes that encode three distinct catalytic subunits (PPP1CA, PPP1CB and PP1CC) which have conserved catalytic cores and variable flanking regions. PP1s interact with a wide range of proteins through interactions with SLiMs, as reviewed in detail elsewhere [180]. In brief, the RVxF motif is the most common PP1 docking motif [181]. It binds to a hydrophobic groove in the catalytic subunit (Figure 4C). Several other PP1 docking motifs have been reported to enable the interactions including the SILK motif, the Rxx[Q/V/I/L K/R]x[Y/W] motif [182], the ΦΦ motif [183] and KiR (Ki67–RepoMan) motifs [184]. Moreover, the binding of RV[S/T]F containing ligands to PP1 during mitosis has been shown to be subjected to phosphorylation by aurora B, which blocks PP1 binding with the phosphorylated regulatory proteins [185]. The phosphorylation by aurora kinases is driven by recognition of the arginine at the p − 2 position [186,187]. The SLiM-binding sites on the catalytic subunit are employed for substrate recognition, for targeting to subcellular compartments and for binding to regulatory domains that in turn recruit substrates [180]. For example, the PDZ protein sphinophilin binds to the PP1A via the RVxF and ΦΦ motifs, which blocks binding of other ligands. Substrates are then targeted to the PP1-sphinophilin complex by PDZ-mediated interactions with C-terminal ligands of target proteins (Figure 4C) [188].

Specificity for PP1 catalytic units among the regulatory domains can be further obtained by auxiliary motif-based interactions involving motifs in the tail regions flanking the catalytic core of the enzyme. Recently, it was shown that TP53BP2 (apoptosis-stimulating of p53 protein 2) binds to the PP1 RVxF site and uses its SH3 domain to discriminate between different PP1 isoforms based on the presence or absence of a PxxPxR motif in the PP1 C-terminal tail [189].

### PP2A

PP2A is a major phosphatase in human cells. It forms a heterotrimeric holoenzyme complex composed of a catalytic subunit (2 subunits), a scaffolding subunit (2 subunits) and a regulatory subunit (15 different subunits of four different classes) [190] (Figure 4B). The determinants of PP2A substrate specificity involves components of subcellular targeting, the combinatorial assembly of the holoenzyme complex and specific interactions with the substrates [190]. The regulatory subunit has a key role in substrate targeting. Compared with the well-defined docking motifs of PP1, the information about the motif-based interactions that contribute to PP2A activity remains scarce. Recently, a [RK]Vxx[VI]R docking motif was described for the B55 regulatory subunit [191]. This motif has been found closely downstream the site of dephosphorylation in substrates such as the retinoblastoma-like protein 1 (RBL1, also called p107) and the microtubule-associated protein tau. Another well-described PP2A docking interaction involves the B56 regulatory subunit. B56 binds to a LxxIxE docking motifs found in the kinetochore protein mitotic checkpoint serine/threonine-protein kinase BUB1 [192], in the nuclear scaffolding protein Repo-Man (cell division cycle-associated protein 2) [193], and in a large number of other proteins as identified by proteomic peptide phage display and bioinformatics analysis [193,194]. The B56-LxxIxE interaction is positively regulated by ligand phosphorylation [192]. Apart from the LxxIxE motif, a subset of ligands have been found to also contain a basic stretch that augments the interaction with LxxIxE containing ligands through interactions with an acidic patch on the B56 structure [195].

### Calcineurin (PP3/PP2B)

Calcineurin is a ubiquitously occurring Ca^2+^/calmodulin-activated serine/threonine phosphatase that links Ca^2+^ signalling to the phosphorylation state of a large number of proteins [196–199]. It is a heterodimer of a catalytic PPP domain (CNA) and a Ca^2+^-binding regulatory domain (CNB). Calcineurin binds to two SLiMs, PIxIxIT and LxVP, of which the PIxIxT motif is the main ligand [196]. It was first discovered in the transcription factor NFAT2 (nuclear factor of activated T-cells, cytoplasmic 2) [197], and has later been found in a large number of proteins. For example, a _86_-PQIIIT-_91_ motif was recently found in the protein spermatogenesis-associated protein 33 [200]. The PIxIxIT motif binds to the catalytic subunit of the CNA [201] and targets the enzyme to substrates and regulators [198]. The LxVP binds to a hydrophobic cleft at the interface of the CNA and CNB subunits that is only available in the active conformation, and may contribute to positioning the phosphorylated residues for dephosphorylation [202,203] (Figure 4C). The presence of both motifs in a substrate may increase the affinity for the substrate through avidity effects, as shown for the calcineurin substrate Na^+^/H^+^-exchanger 1 [204]. Flanking residues and PTMs downstream of the motif also contribute to PxIxIT-CN affinity, as shown by systematic affinity analysis of various ligands [199]. Recently, information on motif-based interactions of calcineurin was largely expanded by a combination of phage-based screening, *in silico* predictions and cell-based approaches [196]. Among the novel findings was that calcineurin is targeted to the centrosomal and nuclear pore complex proteins, and regulates nuclear transport [196].

### PP4

PP4 is a nuclear and chromatin-associated phosphatase [205]. Similar to PP2A, PP4 is often found as a heterotrimeric complex, with a catalytic subunit (PP4c) that forms a complex with a scaffolding subunit (PP4R2) and a regulatory-3 subunit (PP4R3) [177]. The regulatory PP4R3 has an EVH1 (drosophila enabled/vasodilator-stimulated phosphoprotein homologue 1)-like domain that binds to FxxP containing peptides [205,206]. For the drosophila PP4 (falafel) it was shown that the interaction with a FxxP containing motif in the centromeric protein C recruits the enzyme to centromeres [206]. A resource of human PP4 binding motifs was established through a combination of proteomic peptide phage display, bioinformatics and mass spectrometry-based interactomics [205]. It was further shown that the PP4 interaction with a **F**KR**P**T stretch in the coiled-coil domain-containing protein 6 (CCDC6) can be negatively regulated by phosphorylation of the p + 5 position. Modulation of phosphatase docking interactions by phosphorylation may thus be a common theme.

## Outlook

We have here surveyed motif-based interactions that contribute to the specificity of the writer–reader–eraser system of serine/threonine phosphorylation. It is a daunting topic, given the extent of literature. Nevertheless, we expect that the current literature only covers a fraction of all phospho-regulated SLiM-based interactions, and only part of the docking interactions of kinases and phosphatases. Moreover, as showcased here, we would expect that a large proportion of the SLiM-based interactions can be regulated by phosphorylation within the motif or its flanking regions. Much effort will thus be needed to obtain a better understanding of the functional roles of the hundreds and thousands of phosphosites in the proteome, the enzymes that act on them, and what consequences the modification has on SLiM-based interactions. The effects on PTMs on SLiM-based interactions are of course not restricted to phosphorylation, but can be expanded to other PTMs, such as methylation, acetylation, ubiquitination and sumoylation. Along these lines, in the larger scale analysis performed in the switches.elm compendium, it was suggested that more than half of the known validated SLiMs are pre- or post-translationally modulated and regulated [3]. Hence an overall picture can be envisioned in which it is possible that most SLiM-based PPIs are regulated by PTMs or by other means such as pre-translational processing or competition with other proteins.

SLiM-based interactions have previously mostly been identified in low-throughput experiments, but with the use of different display and array-based systems, SLiM-based interactions are now being uncovered on a larger scale [207]. Such large-scale discovery of SLiM-based interactions and binding motifs paired with annotations of known phosphosites allow the predictions of phospho-regulated interactions on a larger scale [70]. Attempts are also being made to elucidate SLiM-based interactions tuned by novel phospho-regulated interactions by a direct experimental approach and on a larger scale. We have for example tested the concept of phosphomimetic proteomic peptide phage display in order to screen for phospho-regulated PDZ binding motifs [151]. In this fashion, we could establish the phospho-regulation of various positions of the PDZ binding motifs relative to the core motif and identify novel interactors [151]. Although useful, it is important to realise that phosphomimetics do not perfectly mimic phosphorylation, and results need to be confirmed using actual phosphorylated peptides [149,151]. Various approaches for higher throughput affinity measurements, such as the hold-up assay [208], FACS-based sorting of yeast-display ligands [209] and spectrally encoded beads [199], are also contributing with information on affinities of SLiM-based interactions and their regulation. The larger scale-motif discovery and paired with higher throughput characterisation will contribute towards a more detailed picture of the SLiM-based molecular interactions that govern cell function, and the interplay between the writers, readers and erasers of the phosphorylation code.

## Abbreviations

BRCT: BRCA1 C-terminal
CAMKK2: calcium/calmodulin-dependent protein kinase kinase-2
CDKs: cyclin-dependent kinases
DOCK1: dedicator of cytokinesis protein 1
DUSPs: dual-specificity phosphatases
FHA: forkhead-associated
GUK: guanylate kinase
JNK1: Jun N-terminal kinase 1
MAGUKs: membrane-associated guanylate kinases
MKKs: MAPK kinases
mTOR: mechanistic target of rapamycin
mTORC1: mTOR complex 1
MYBL2: myb-related protein B
NLSs: nuclear localisation signals
OXSR1: oxidative stress response kinase
PBD: polo-box domain
PDK1: phosphoinositide-dependent kinase-1
PIF: PDK1-interacting fragment
PIN1: peptidyl-prolyl isomerase 1
PLKs: polo-like kinases
PP1: protein phosphatase 1
PPMs: metal-dependent protein phosphatases
PPPs: phosphatases: phosphoprotein phosphatases
PTMs: post-translational modifications
RBL1: retinoblastoma-like protein 1
RSKs: ribosomal S6 kinases
SLiMs: short linear motifs
SPAK: ste20-related proline alanine-rich kinase
TOS: TOR signalling
